# Study Protocol – Metabolic syndrome, vitamin D and bone status in South Asian women living in Auckland, New Zealand: A randomised, placebo-controlled, double-blind vitamin D intervention

**DOI:** 10.1186/1471-2458-8-267

**Published:** 2008-07-31

**Authors:** Pamela R von Hurst, Welma Stonehouse, Christophe Matthys, Cathryn Conlon, Marlena C Kruger, Jane Coad

**Affiliations:** 1 Institute of Food, Nutrition and Human Health, Massey University, Auckland, New Zealand; 2 Institute of Food, Nutrition and Human Health, Massey University, Palmerston North, New Zealand

## Abstract

**Background:**

The identification of the vitamin D receptor in the endocrine pancreas suggests a role for vitamin D in insulin secretion. There is also some limited evidence that vitamin D influences insulin resistance, and thus the early stages of the development of type 2 diabetes.

**Methods:**

Eighty-four women of South Asian origin, living in Auckland, New Zealand, were randomised to receive either a supplement (4000IU 25(OH)D_3 _per day) or a placebo for 6 months. At baseline, all participants were vitamin D deficient (serum 25(OH)D_3 _<50 nmol/L), insulin resistant (HOMA-IR > 1.93) and/or hyperinsulinaemic, hyperglycemic or had clinical signs of dislipidaemia. Changes in HOMA-IR, lipids, parathyroid hormone, calcium and bone markers were monitored at 3 months and 6 months.

**Discussion:**

This randomised, controlled trial will be the first to investigate the effect of vitamin D supplementation on insulin resistance in non-diabetic subjects. It will subsequently contribute to the growing body of evidence about the role of vitamin D in metabolic syndrome.Registered clinical.

**Trial registration:**

Registered clinical trial – Registration No. ACTRN12607000642482

## Background

Hypovitaminosis D is becoming recognised as a worldwide problem, and exists even in countries such as New Zealand and Australia which enjoy plentiful sunshine and latitudes sufficiently moderate to allow some endogenous synthesis throughout the year [1-5]. Once thought to impact only on bone health, there is now evidence to implicate vitamin D deficiency in a plethora of adverse health conditions such as cancer and auto-immune diseases. There is mounting interest in the role of vitamin D in the constellation of metabolic abnormalities grouped under the term "metabolic syndrome", including hypertension, dyslipidaemia, abdominal obesity, glucose intolerance and type 2 diabetes.

Low serum vitamin D has been shown to correlate with impaired glucose tolerance [1,6-8], whilst administration of supplemental vitamin D to subjects with elevated blood glucose levels has resulted in an improvement in insulin secretion [9,10]. Similar improvements have been observed in vitamin D-deficient subjects following supplementation [6,11].

The role, if any, that vitamin D deficiency plays in insulin resistance has had little investigation. Whilst a correlation between hypovitaminosis D and insulin resistance has been identified in pregnant women [12] and obese adolescents [13] randomised, controlled trials with vitamin D supplementation are sparse, especially in non-diabetic, insulin resistant, vitamin D deficient subjects. Borissova et al [10] measured a 29% (but not significant) decrease in HOMA-IR following supplementation of 1332IU 25(OH)D_3_/day for one month in a group of 10 diabetic women, 70% of whom were vitamin D deficient (<50 nmol/L). Pittas et.al. [14] saw a significantly lower increase in HOMA-IR in vitamin D/calcium supplemented subjects with impaired fasting glucose, compared to those receiving calcium only, but subjects were not vitamin D deficient. Meanwhile, Taylor [15] reported an increase in insulin resistance in 3 diabetic patients following a single intramuscular dose of 300,000 IU of 25(OH)D_2_.

The importance of vitamin D in the maintenance of the calcium economy and bone health is well known. A significant positive correlation has been found between serum vitamin D levels and bone mineral density in South Asians living in India [16], Britain [17] and Bangladesh [18]. An inverse relationship has been identified between vitamin D status and OC, a marker of bone turnover [19]. Bone markers are useful for identifying changes in bone metabolism, particularly when monitoring intervention or therapy [20].

The population of interest in this study is women of South Asian origin living in Auckland, New Zealand. There is a high incidence of type 2 diabetes in South Asians living in New Zealand; self-reported diabetes is over three times higher for this group compared to national incidence [21]. The possibility of an ethnic or genetic predisposition to develop diabetes in this population is supported by epidemiological evidence from around the world [22] and India itself where there are over 33 million people diagnosed with diabetes [23].

Between 1991 and 2001 the numbers of Asian Indians living in New Zealand doubled. This trend has continued exponentially with a current population of over 113,000 South Asians living predominantly in the Auckland area [24]. The projections for future growth indicate that the Asian population of which Indians are the second largest group will increase from 272,000 in 2001 to 667,000 by 2021, an increase of 395,000 (145%) [24].

Very little is known about vitamin D status of this population. However, a number of studies suggest that South Asian migrants may have a higher risk of disease states associated with low vitamin D status such as infantile rickets, osteoporosis and both type-1 and type-2 diabetes [22,25,26]. Reasons for low vitamin D status are various and mostly unconfirmed. They include low incidental exposure to sunlight, deliberate avoidance of sunlight, limited dietary intake of vitamin D (there is no routine fortification of the New Zealand food supply with vitamin D) and possibly genetic factors. Polymorphisms in the vitamin D receptor, which are thought to modify susceptibility to diabetes, have been identified in both Indian and Bangladeshi populations [27].

### Hypotheses

• There is a high prevalence of hypovitaminosis D in South Asian women living in Auckland, New Zealand.

• Supplementation of vitamin D in subjects who have demonstrated insulin resistance and vitamin D deficiency will result in an improvement in markers for metabolic syndrome.

• Supplementation of vitamin D in subjects who have demonstrated vitamin D deficiency will result in an improvement in bone marker ratios in favour of bone mineralisation.

### Aims

• To establish the vitamin D status of South Asian women living in Auckland, NZ.

• To investigate the effectiveness of vitamin D supplementation in improving insulin sensitivity in women with hypovitaminosis D who have demonstrated insulin resistance, and/or lipid profiles in those with dyslipidaemia.

• To investigate the effect of vitamin D supplementation on bone marker ratios in women who have hypovitaminosis D.

## Method

**Figure 1:**
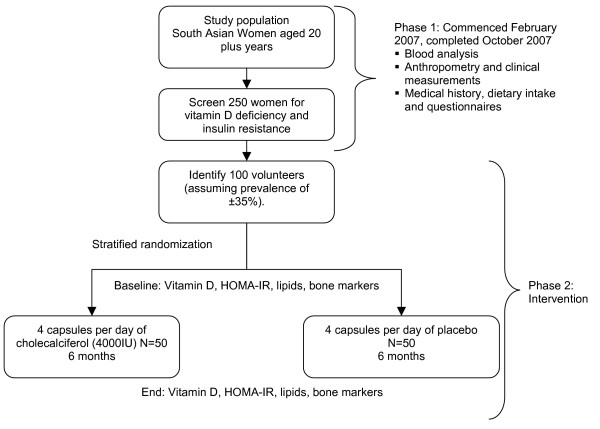
Study design of the SURYA Study.

The study consists of two phases: Phase one included initial recruitment and screening, phase two included recruitment of selected individuals into the vitamin D intervention trial (Figure 1).

Phase one provided the opportunity for a comprehensive description of the population group with respect to general health, anthropometry, diet and other lifestyle factors. The majority of the phase one data were collected during one visit of the participant to either the Massey Human Nutrition Research Unit or Mount Roskill Surgical and Medical Centre. Participants were weighed and measured, blood samples taken, blood pressure and aural temperature measured, then the participant was given breakfast. During breakfast the medical history form was completed and checked and the diet diary explained.

### Participants

It was calculated that 42 subjects would be required for each arm of the trial to demonstrate a significant difference at 80% power and 5% significance. Power calculations were based on the results of a lifestyle intervention which achieved a reduction in HOMA-IR of 0.98 [28], and derived from Margetts and Nelson [29].

Women were invited to participate in the study through advertisements in suburban and Auckland newspapers, and Indian media such as television, radio and newspapers. Posters and leaflets were distributed in a number of venues around Auckland such as General Medical Practices with high numbers of South Asian patients, clubs, and temples. A number of South Asian social and community groups were given presentations about the study by the researchers.

A short television documentary on a channel popular with Indian viewers elicited the greatest response of all the methods used. However, the most effective form of promotion was word-of-mouth. A relatively small group of supportive women from the South Asian community promoted the study and were instrumental in the successful recruitment of 250 women.

### Inclusion/exclusion criteria

Women had to be 20+ years and of South Asian origin; either the subject, both parents or all grandparents must have been born on the Indian sub-continent i.e India, Pakistan, Bangladesh, Sri Lanka – generally referred to as South Asia.

Volunteers were excluded if suffering from significant renal dysfunction, major systemic illness, or diabetes requiring medication. Use of 25(OH)D_3 _supplements exceeding 1000 IU/day (i.e. prescription dose), or any form of calcitriol (1, 25(OH)_2_D_3_) were also exclusion criteria.

Additional inclusion criteria for the intervention trial were low vitamin D (<50 nmol/L) and a HOMA-IR (insulin resistance) measurement of >1.93 or hyperinsulinaemia (FPI >13 mIU/L) or hyperglycaemia (FPG 5.6 – 7.2 mmol/L) or a triglyceride/HDL-C ratio >3.0. HOMA-IR >1.93 was based on the upper quartile determined in the Chennai Urban Population Study-7 [30]. A triglyceride/HDL ratio >3.0 has been shown to be a surrogate marker for early insulin resistance in overweight/obese people [31].

### Funding and ethics

Funding for the study was provided by the New Zealand Lottery Board (Lottery Health Grant), New Zealand Department of Internal Affairs. Additional support was provided by Blackmores Pty Ltd, Australia, who supplied the active supplement and the placebo.

Ethical approval was granted by the Massey University Human Ethics Committee (Southern A), Reference No. 06/67 and the subjects signed an informed consent form for participation in the study.

### Setting

The study took place in Auckland, New Zealand. Auckland is New Zealand's largest city with a population of just over 1 million. The majority of South Asian immigrants settle in Auckland [24].

### Vitamin D intervention

Initial screening (phase 1 of the study) took place over a period of 9 months, commencing February 2007. See Table 1 for outcome measures and testing methods for phase 1. From the 250 women screened in phase 1, 116 qualified for phase 2, with the inclusion criteria described above. Women with FPG >7.2 mmol/L were offered a second blood test, and then withdrawn from the study and referred to their medical practitioner if the second blood test yielded similar results.

**Table 1:** Outcome measures and testing methods used for phase one.

**Measure**	**Method**
**Blood analysis**	All analysed by LabPlus, Auckland City Hospital, Auckland
Fasting plasma insulin	Micro-particle enzyme immunoassay technology (MEIA), Abbott Diagnostics
Fasting plasma glucose	An enzymatic colourimetric assay; Roche Glucose reagent kit (Cat. No. 1876899)
Total cholesterol	Enzymatic colourimetric method (Roeschlau and Allain). Roche Cholesterol reagent kit (Cat. No. 1491458)
Triglycerides	Enzymatic conversion. Roche Triglycerides reagent kit (Cat. No. 1727711)
HDL-C	Homogenous enzymatic colourimetric assay. Roche HDL-Cholesterol Plus reagent kit (Cat. No. 04713214)
LDL-C	Calculated
Serum calcium	Colourimetric assay. Roche Calcium reagent kit (Cat. No. 1730240)
Serum albumin	Colourimetric assay. Roche Albumin Plus reagent kit (Cat. No. 1970909)
Serum high sensitivity CRP	Particle enhanced immunoturbidimetric assay. Roche CRPLX reagent kit (Cat. No. 03002039)
Serum parathyroid hormone	"ECLIA" electrochemiluminescence immunoassay. PTH Reagent Pack (Cat. No. 11972103122)
Serum 25 OH vitamin D	Radioimmunoassay, DiaSorin RIA kit (Cat. No. 68100E)
**Anthropmetric and clinical assessments:**	
Blood pressure	Omron HEM-907 Digital Automatic Blood Pressure Monitor
Temperature	Braun ThermoScan Pro 3000 aural thermometer
Height, weight, waist and hip circumference	ISAK anthropometry methods – ISAK level one accredited anthropometrist, Tanita electronic scales, stadiometer, Lufkin tape
**Questionnaires:**	
Dietary assessment	4-day food diary. Completed by subjects. Analysed using Foodworks 2007 (Xyris Software). Additional recipes and food lists provided by Indian dietitian
Medical and family history	Questionnaire administered following blood tests
Demographics	

One hundred and six women were recruited for phase 2, but a number were lost to the study due to becoming pregnant (n = 3), moving overseas (n = 6) perceived side-effects (n = 2), medical practitioner prescribing vitamin D (n = 3), or withdrew from study (n = 8).

The intervention consisted of a 25(OH)D_3 _supplement, 4000 international units (IU) per day, or placebo, in the form of 4 oral capsules, for 6 months. The consumption of 4000 IU 25(OH)D_3 _has been shown to be a safe and effective dose [32,33]. The active supplement was tested for 25(OH)D_3 _content by the Massey University Nutrition laboratory. The average content per dose was 4226 IU, no vitamin D was detected in the placebo. The vitamin D content will be tested again at the completion of the study to determine any degradation during storage over the period of the study.

Subjects were matched into pairs by age and BMI. Randomisation of the active/placebo capsules and allocation to pairs was performed by Blackmores using nQuery Advisor^®^, version 6.0, (Statistical Solutions, Cork, Ireland). Randomisation and allocation were fully concealed from the researchers.

Once recruited into phase two, participants were recalled for a baseline blood test prior to being given their supply of supplements. After 3 months another blood sample was taken to check for hypercalcaemia. Once the intervention was completed (6 months) participants provided one more blood sample and were weighed again. See Table 2 for outcome measures and testing methods for the intervention phase of the study.

**Table 2:** Outcome measures and testing method for phase two (measured at baseline, 3 months and 6 months)

**Test**	**Method**
Blood tests As listed in table 1, plus:	
Collagen C-telopeptide (CTX) Osteocalcin (OC)	Roche Elecsys beta cross-laps Roche Elecsys 2010 Canterbury District Health Board Laboratory
Weight at baseline and 6 months	Tanita electronic scales

### Blood sampling and processing

Venous blood samples were taken by registered phlebotomists, using a sterile Vacutainer Flashback needle and needle holder between 8.30 am and 9.30 am. There is considerable circadian variability in bone markers; OC levels are increased by 20% at peak (very early morning) and CTX levels at the nadir may be half those at the nocturnal peak [20]. Accordingly, all blood samples were obtained within a consistent time period. The subjects were asked to fast overnight (at least 8 hours with no food or beverage, excluding water). Serum was used for the analysis of lipid profiles, CRP, calcium, albumin, parathyroid hormone, glucose, insulin, vitamin D. The blood was protected from light, allowed to clot for ± 30 minutes and centrifuged for 10 minutes at 2000 g at 4°C within 2 hours. For the preparation of plasma, the blood was collected in vacutainers buffered with EDTA anticoagulant, and centrifuged for 10 minutes at 2000 *g *at 4°C. For OC and CTX, plasma was dispensed and frozen within 2 hours of collection and flown to Christchurch, New Zealand on dry ice for analysis at conclusion of the study. Aliquots of serum and plasma were stored at -80°C whilst awaiting analysis.

### Questionnaires

The medical history form was completed by participants during their visit for blood tests and anthropometry. Before the participant departed the researcher checked all answers and probed any responses requiring clarification. The questionnaire included basic demographics, country of birth, length of time in New Zealand and number of years of education since age 5 years. All medication and dietary supplement use was recorded, together with tobacco, betel nut and alcohol use, menstrual status, birth control and/or HRT use, and dental history. Family history of diabetes, CVD and osteoporosis was also investigated.

The 4-day estimated dietary record was presented in an open entry booklet form with pages for recording each day's food intake and extra pages for recipes. Participants were asked to record at least one weekend or feast day. Measuring cups and spoons were provided if required. Instructions for the completion of the diary were given verbally when the participant received the diary and a list of written instructions, including examples, were provided on the first 2 pages of the booklet. The women were asked to give a detailed description of the foods eaten, if possible to give a brand name and to estimate the amounts using natural measures (e.g., pieces, slices) or household measures (e.g., coffee spoon, cup). Experienced dietitians used a standardised protocol, including a manual on household weights and measures to convert the estimated amounts into weights. All dietary records were checked for quality and completeness prior to data entry into the Foodworks 2007 programme. Additional new foods and recipes were added as required, with food composition data taken from an Indian food composition reference [34]. The recipes were adapted for individual subjects based on the ingredients that are being used in New Zealand. After data entry, each record was verified by a nutrition researcher and corrections were made if mis-interpretation or errors in data entry were found. To ensure the best possible quality of dietary data, a subset of subjects were recalled for a qualitative, in-depth interview with a dietitian assessing their 4-day food records for dietary content (quality and quantity), food types, additions to foods and drinks and recipe composition (to further clarify fat and sugar intake.)

In the present study, a 4-day estimated dietary record was chosen because of the high respondent burden and time-consuming characteristics of 7-day dietary food records. However, Nelson *et al *[35] stated that a 4-day record, randomised to cover weekday variations, seems to be the optimum. To obtain a highly accurate estimate of individual intake, a longer period would be recommended for many nutrients, especially those with a high within-person variation, but this was not feasible due to practical considerations [36].

Participants were given a free-post, pre-addressed envelope for the return of the booklet.

### Provision of results to participants

Following the receipt and analysis of the 4-day diet diary and the completion of the biochemical assays done during the screening phase, each participant received a feedback form. This contained an overview of the analysis of the diet, with particular reference to macronutrients, iron and calcium. Anthropometric measurements, blood pressure and blood results (fasting glucose, fasting insulin and lipids) were also included. Once recruitment into the trial was completed, participants not involved in the trial received notification of their vitamin D levels together with advice about supplementation where applicable.

On completion of the trial, participants will be informed of their current vitamin D status, their baseline status and if they were taking the active or placebo dose. All trial participants will be given 6 months supply of vitamin D supplements (courtesy of Blackmores), regardless of which arm of the trial they were on, upon completion of the 6-month intervention.

Newsletters were sent to all participants at intervals during the study with the goal of keeping them interested and informed.

### Data Handling and statistical analysis

Name and address details were maintained in Microsoft Access. Check boxes recorded the progress of a participant through the study and thus allowed personalised letters to be sent to groups of participants as required throughout the study. All other data were entered into a single Microsoft Excel spreadsheet with participants identified only by their unique Subject Number. All entries were double-checked by another member of the research team.

Before commencement of statistical analysis the data will be cleaned and checked for coding errors. Descriptive statistics will be used for the baseline population characteristics: mean (SD), median, mode and range summary statistics, independent t-tests and paired t-tests for numerical normally distributed data, and Mann Whitney U and Wilcoxon Paired Rank Test (for paranormal distribution) and Chi-Squared tests (for categorical data). Spearman (checked with Pearson) correlations and ANOVA will be calculated to test for associations between variables.

The primary analysis will be a comparison of the change of the primary outcome (vitamin D status) between the intervention and the control group following the 'intention to treat' principle at three months and six months. In order to assess whether protocol deviations have caused bias, the results of the intention-to-treat analyses will be compared to 'per protocol' analyses. Furthermore secondary analyses will be performed to explore intervention effects on the insulin resistance, HDL/triglycerides and bone markers. Analysis of covariance will be used to analyse the effects of treatment on changes in variables while controlling for the effects of possible confounding factors for example different baseline conditions (e.g. BMI). Statistical analyses will be performed by using SPSS software (version 15).

## Discussion

The information that was obtained from the screening phase of this study provided a valuable and unique insight into this rapidly increasing group of migrants entering New Zealand. It was not the intention during this phase to conduct a cross-sectional study and there was no attempt to recruit in a fully randomised way. Indeed, such recruitment in any specific, ethnic minority within a large population would be highly problematic. However, the 250 women who participated in phase one of the Surya Study represent approximately 0.5% of the total number of South Asian women living in New Zealand. Distribution in terms of country of origin reflected that of the entire population as reported in the 2006 census [24]. Level of education, although higher than the New Zealand national average, was as expected of a newly migrant population given New Zealand's immigration requirements for high educational standards [37].

As far as we are aware, there has been no other randomised, placebo-controlled, double-blind trial investigating the effect of vitamin D supplementation on insulin resistance and other markers of metabolic syndrome. If this trial is successful, it will provide another clue about the aetiology of type 2 diabetes, and the opportunity to reduce one of the many risk factors.

## Abbreviations

BMI: body mass index; CTX: Collagen C-telopeptide; CVD: cardiovascular disease; EDTA: ethylene diamine tetraacetic acid; FPG: fasting plasma glucose; FPI: fasting plasma insulin; HOMA-IR: homeostasis model assessment insulin resistance; HRT: hormone replacement therapy; IU: international units; OC: osteocalcin; PTH: parathyroid hormone.

## Competing interests

The authors declare that they have no competing interests.

## Authors' contributions

PRvH and JC conceived the study, acquired funding and ethics approval. PRvH coordinated recruitment, participant management and data collection. WS and CC designed the CVD risk section and the laboratory protocol. MCK advised on the bone section. CM advised on statistical analysis. PRvH drafted the manuscript. All authors were involved in revising the manuscript and all read and approved the final manuscript.

## Pre-publication history

The pre-publication history for this paper can be accessed here:


